# Evidence of Disruption in Neural Regeneration in Dry Eye Secondary to Rheumatoid Arthritis

**DOI:** 10.3390/ijms24087514

**Published:** 2023-04-19

**Authors:** Balázs Sonkodi, Anita Csorba, László Marsovszky, Attila Balog, Bence Kopper, Zoltán Zsolt Nagy, Miklós D. Resch

**Affiliations:** 1 Department of Health Sciences and Sport Medicine, Hungarian University of Sports Science, 1123 Budapest, Hungary; 2 Department of Ophthalmology, Semmelweis University, 1085 Budapest, Hungary; 3 Department of Rheumatology and Immunology, Faculty of Medicine, Albert Szent-Györgyi Health Center, University of Szeged, 6725 Szeged, Hungary; 4 Faculty of Kinesiology, Hungarian University of Sports Science, 1123 Budapest, Hungary

**Keywords:** dry eye disease, rheumatoid arthritis, autoimmune disease, Piezo2 channelopathy, K_2P_ channel, TASK1 channel

## Abstract

The purpose of our study was to analyze abnormal neural regeneration activity in the cornea through means of confocal microscopy in rheumatoid arthritis patients with concomitant dry eye disease. We examined 40 rheumatoid arthritis patients with variable severity and 44 volunteer age- and gender-matched healthy control subjects. We found that all examined parameters were significantly lower (*p* < 0.05) in rheumatoid arthritis patients as opposed to the control samples: namely, the number of fibers, the total length of the nerves, the number of branch points on the main fibers and the total nerve-fiber area. We examined further variables, such as age, sex and the duration of rheumatoid arthritis. Interestingly, we could not find a correlation between the above variables and abnormal neural structural changes in the cornea. We interpreted these findings via implementing our hypotheses. Correspondingly, one neuroimmunological link between dry eye and rheumatoid arthritis could be through the chronic Piezo2 channelopathy-induced K_2P_-TASK1 signaling axis. This could accelerate neuroimmune-induced sensitization on the spinal level in this autoimmune disease, with Langerhans-cell activation in the cornea and theorized downregulated Piezo1 channels in these cells. Even more importantly, suggested principal primary-damage-associated corneal keratocyte activation could be accompanied by upregulation of Piezo1. Both activation processes on the periphery would skew the plasticity of the Th17/Treg ratio, resulting in Th17/Treg imbalance in dry eye, secondary to rheumatoid arthritis. Hence, chronic somatosensory-terminal Piezo2 channelopathy-induced impaired Piezo2–Piezo1 crosstalk could result in a mixed picture of disrupted functional regeneration but upregulated morphological regeneration activity of these somatosensory axons in the cornea, providing the demonstrated abnormal neural corneal morphology.

## 1. Introduction

Rheumatoid arthritis (RA) is one of the most frequent chronic autoimmune diseases, with synovial inflammation and bone erosion features. The etiology of RA is not entirely known, with associated genetic and environmental risk factors, but it involves both the innate and adaptive immune systems in inflammatory processes that also pertain to activation of immune-complex-mediated complements and overproduction of proinflammatory cytokines [1].

Dry eye disease (DED) is a complex multifactorial and heterogeneous condition of the ocular surface, characterized by symptoms of ocular discomfort and irritation. Despite significant progress in our understanding of this debilitating disease, its precise etiology is yet to be elucidated [2,3,4]. Interestingly, DED was mainly discussed in relation to autoimmune diseases more than three decades ago [3]. Correspondingly, it is known that dry eye (DE) is one extra-articular manifestation of RA [5].

Nobel laureate Ardem Patapoutian and his team identified the Piezo2 ion channel as the principal ion channel responsible for proprioception [6]. It has been theorized recently that initial primary microdamage in autoimmune diseases could be autologous proprioceptive terminal Piezo2 microdamage that could evolve into a chronic disease condition due to chronification of the Piezo2 channelopathy, with underlying genetic and environmental risk factors [7,8]. Correspondingly, current authors theorize that the primary damage in RA could also be related to microdamage of Piezo2-containing primary-joint-afferent terminals that contribute to proprioception. Indeed, one study already indicated that cervical proprioception is impaired in RA [9]. Moreover, findings about the role of the joint–brain axis and the crosstalk between chronic peripheral inflammation and the brain in RA are emerging [10]. Interestingly to note, comparative genome-wide association studies found 15 single-nucleotide polymorphisms (SNPs) commonly linked to RA and frontotemporal dementia, but shared disease-associated SNPs also link RA to amyotrophic lateral sclerosis (ALS) [11]. The primary damage of these neurodegenerative diseases is theorized to be irreversible Piezo2 microinjuries at proprioceptive sensory terminals [12,13,14]. Even very recently, the CACNA1D gene of the Ca_v_1.3 ion channel was found to be a likely susceptibility gene of ALS [14], as it is in RA [15], and this susceptibility gene could contribute to miswired proprioception [14] and lead to altered neurotransmitter signaling along the joint–brain axis [10]. Furthermore, this noncontact Piezo2 microinjury mechanism was also proposed for DED [16]. Thus, the question of whether a link between autoimmune diseases and DE could exist seems to be appropriate.

There is an unmet need to further explore the potential role of the corneal neuroimmune mechanism in development of DED in autoimmune diseases, such as in RA. In brief, we theorize that chronic Piezo2 channelopathy on corneal somatosensory terminals could lead to lost functional remodeling and regeneration capacity of the cornea, and as a result, the morphology could show an abnormally diminished regenerative picture of nerve fibers in DE secondary to RA. Therefore, our aim was to investigate signs of reduced regeneration activity in corneal sensory afferents using confocal microscopy.

## 2. Results

The demographics and clinical data of patients are summarized in Table 1. No significant difference was found in the age or gender distribution. All dry-eye parameters indicated DED; the Schirmer test results and the tear break-up time (TBUT) were lower, while the ocular-surface disease index (OSDI) score showed a higher value in the RA group compared to the controls.

**Figure 1:**
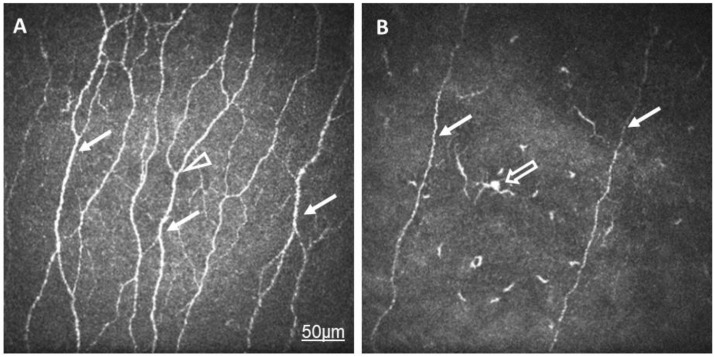
In vivo confocal microscopic images of corneal subepithelial nerves in a healthy eye (**A**) and in the eye of a patient with rheumatoid arthritis and dry eye (**B**). Note the lower density and reduced thickness of the subepithelial nerve plexi (arrow) in RA compared to in normal eyes. The number of branches (arrowhead) is minimal in RA. Langerhans cells with elongated dendrites are also demonstrated (empty arrow).

Confocal corneal microscopy of the subepithelial layer clearly demonstrated a significant decrease in corneal subepithelial nerve plexi. Not only the density but the thickness of the nerves and the number of branches revealed damaged nerve structure (Figure 1).

### 2.1. Comparison of RA and Control Groups

For all variables, such as corneal nerve-fiber density (CNFD, the number of fibers/mm^2^), corneal nerve-branch density (CNBD, the number of branch points on the main fibers/mm^2^), corneal nerve-fiber length (CNFL, the total length of the nerves in mm/mm^2^), corneal nerve-fiber total branch density (CTBD, the total number of branch points/mm^2^), corneal nerve-fiber area (CNFA, the total nerve-fiber area in mm^2^ per mm^2^) and comparison of the RA and the control samples resulted in significant differences (*p* < 0.05). In all of the cases, the RA-sample means were significantly lower than in the control samples (Table 2).

### 2.2. Correlation Analysis

**Figure 2:**
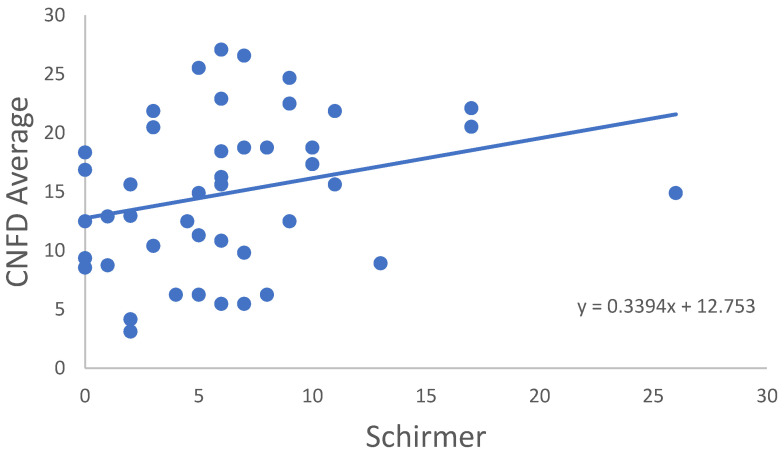
Correlation of CNFD Average and Schirmer’s-test results.

**Figure 3:**
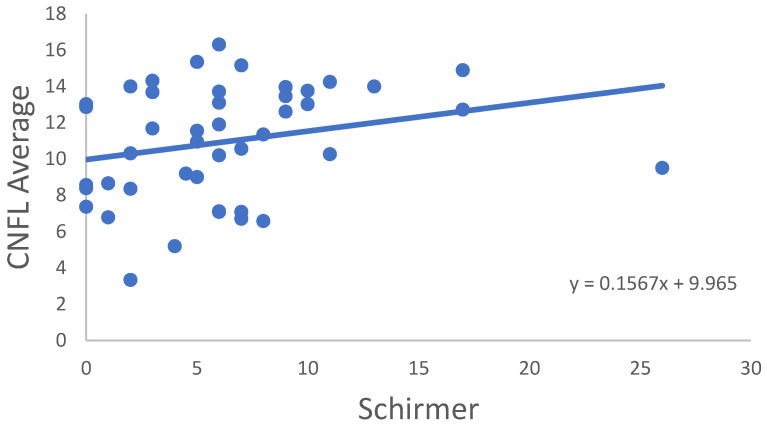
Correlation of CNFL Average and Schirmer’s-test results.

**Figure 4:**
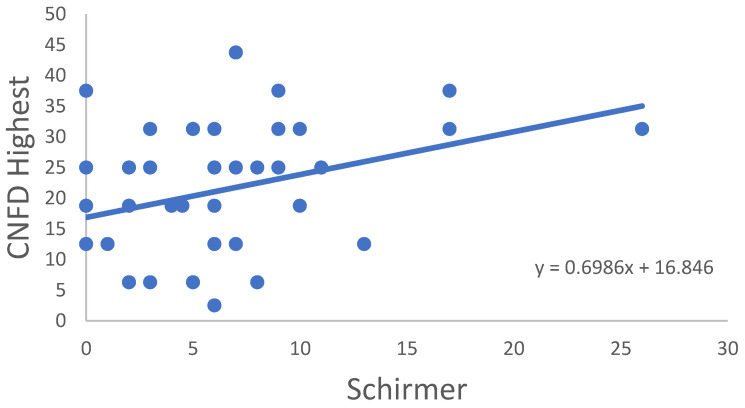
Correlation of CNFD Highest and Schirmer’s-test results.

**Figure 5:**
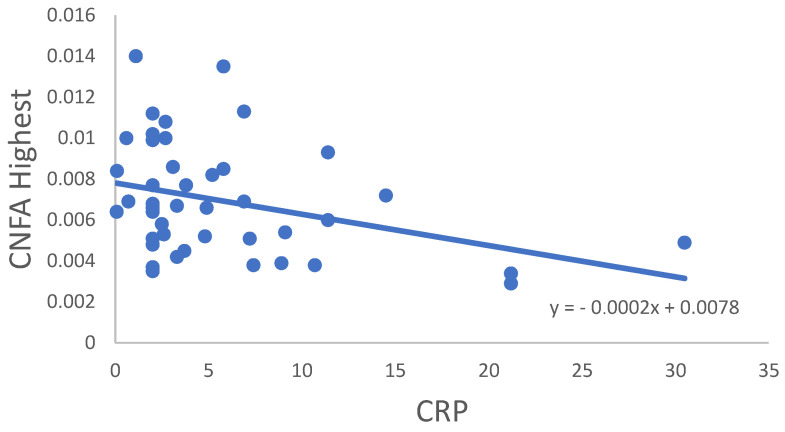
Correlation of CNFA Highest and CRP values.

In the RA group, correlation of all CNFD–CNBD–CNFL–CTBD–CNFA variables was not significant in age, disease onset, disease activity score (DAS28) or TBUT or OSDI score. In some cases, significant correlations were observed between the variables for the RA samples (Table 3). CNFD Average (Figure 2), CNFL Average (Figure 3) and CNFD Highest (Figure 4) were in significant correlation with the Schirmer values. A significant negative correlation of CNFA Highest was found with the CRP values (Figure 5). It must be noted that although the *p* values indicated statistical significance, the correlation coefficient values suggested only moderate correlation.

Multivariate correlations were also performed in order to detect complex relations among systemic descriptives and dry-eye parameters as well as corneal nerve structural changes, but no significant correlation could be detected.

### 2.3. Subgroup Analysis

Several subgroups were created in the RA group, with the aim to identify influencing factors of corneal nerve changes in RA. No difference was found between male and female patients. Age did not have any effect on the changes; the subgroups of patients under and over 50 years had no difference. Disease duration did not affect corneal nerve structures either; no difference was found between patients with less than and more than 10 years after disease onset. The corneal nerve parameters in the subgroups determined by the DAS28 score to be under and over 2.6 had no significant difference.

## 3. Discussion

Our study found that corneal nerve structure was significantly altered in our RA-related DE subjects. We found evidence of disrupted regeneration activity due to neural damage in the form of decreases in the number, density and length of the sub-basal nerves, along with decreased density of corneal branching points. The current authors suggest that this disruption in regeneration of nerve fibers could have been the result of impaired crosstalk between corneal somatosensory-terminal Piezo2 and peripheral-cell Piezo1 due to chronic Piezo2 channelopathy.

Noteworthily, Piezo ion channels facilitate the mechanogating feature of K_2P_ channels [17], and K_2P_ channels have a well-known role in autoimmune attacks in the central nervous system and in neurodegeneration through the TWIK-related acid-sensitive K^+^ channel 1 (TASK1) pathway [18]. Moreover, activated T lymphocytes are TASK1-dependent [19] with a suspected role in T cell-driven autoimmunity [20,21]. The current authors propose that Piezo2 channelopathy-induced K_2P_ channel activation through TASK1 channels on the spinal level could promote one pathomechanistic link between autoimmune diseases and DE. An indicative finding of this theory is that K_2p_5.1 channel expression in CD4^+^ T lymphocytes in the peripheral blood correlates with disease activity in RA [22].

We presented earlier that corneal Langerhans cells (LCs), a type of dendritic cell, are activated in RA and may play a central regulator role in local corneal immune responses through activation of the innate immune system [5]. Noteworthily, autoimmune diseases are suggested to show a trajectory where CD3^+^/CD56^+^ natural killer-T cell (NKT cell) levels are elevated in the initial stage of the disease process as a first-line cellular innate response but NKT cells are depleted in later stages [8]. Indeed, NKT cells are also depleted in RA [23]. It is postulated that heat shock protein 70 (Hsp70) under pathological hyperexcitation activates the toll-like receptor 4 (TLR4)/Interleukin-6 (IL-6)/tumor necrosis alpha (TNF-α) pathway, leading to an increase in NKT cells in the acute stage of Piezo2 channelopathy [24]. It has been demonstrated in another autoimmune disease that Hsp70 overly activates dendritic cells at the beginning phase of the disease [25] and induces excessive TNF-α and Interleukin-17 (IL-17) release from these dendritic cells, with TLR4/nuclear factor-kappa B (NF-κB) pathway participation [8,26,27,28,29]. Accordingly, Hsp70 is shown to elevate CD4^+^IL-17^+^ (Th17) T helper cell frequencies and shift Th17/T regulatory (Treg) balance, while CD4^+^IFN-γ^+^ (Th1) T helper cells decrease as the/ratio between Th1 and CD4^+^IL-4^+^ (Th2) decreases in an autologous way through IL-6 modulation in RA [1]. Indeed, the balance and plasticity of the Th17/Treg ratio through IL-6 is essential to maintain immune homeostasis and to prevent the autoimmune response in RA [30]. Hence, Piezo2 channelopathy on somatosensory terminals contributing to proprioception could not only activate the innate immune system through IL-6 but also activate the adaptive immune system in RA. Accordingly, Piezo2 channelopathy, which has been suggested to be associated with IL-6 increases through the Hsp70/TLR4/Myd88/IL-6 pathway, as in DOMS [31], could lead to an imbalanced ratio of Th17/Treg in RA, therefore paving the way to the autoimmune response.

Tocilizumab, an IL-6 antagonist monoclonal antibody, reduces IL-33 levels in RA [32]. The role of the IL-31/IL-33 axis in autoimmune diseases, including RA, could be familiarized from the excellent review of Murdaca et al. [33]. That review highlighted the pathways of how IL-33, a tissue-damage-derived nuclear cytokine in, e.g., fibroblast-like cells, could activate Tregs as part of the IL-1 family, leading to crosstalk with the IL-6 pathway downstream through Myd88 and eventually to activation of NF-κB. Indeed, elevated IL-33 concentration in serum and synovial fluid also means increased RA disease activity [34]. The current authors suggest that Tocilizumab could decrease proprioceptive terminal Piezo2 channelopathy-induced IL-6 elevation; thus, it could inhibit crosstalk with the activated IL-31/IL-33 axis through Myd88 and activation of NF-κB. Hence, Tocilizumab could prevent activation of the RA disease process and, as a result, could highlight the critical role of primary joint-afferent-terminal Piezo2 channelopathy participation in RA.

It is important to note that Fernandez-Trillo et al. highlighted the special genetic-signature feature of corneal Piezo2-containing somatosensory fibers in the cornea [35]. Furthermore, it was recently suggested that the primary microinjury of Piezo2 on proprioceptive terminals could activate transcription pathways [13,14] that are the bases for the activated growth, regeneration and remodeling processes [36]. A problem could arise when underlying genetic mutations or environmental risk factors impede the due closure of this activated transcription process, resulting in an unfinished healing process and chronic Piezo2 channelopathy instead of a transient process [7,16].

Notably, painless primary noncontact somatosensory Piezo2 terminal microdamage is suspected to impair intimate crosstalk between the somatosensory Piezo2- and Piezo1-channel-containing peripheral cells in the cornea [16]. Since the cornea contains a delicate network of corneal sensory terminals, corneal dendritic cells and corneal keratocytes, their intimate and continuous interaction is of paramount importance to maintain homeostasis. This theory could be analogous to impaired crosstalk between axonal Piezo2 and Piezo1 of oligodendrocytes in the central nervous system in multiple sclerosis (MS) [37]. Similarly, we hypothesize that impaired Piezo crosstalk causes Piezo1 downregulation in activated corneal LCs in RA-associated DE, as it does in oligodendrocytes in MS [37]. Moreover, the current authors postulate that Piezo1 in activated corneal keratocytes could be upregulated in a feed-forward manner in DED, as was suggested in psoriasis [8], due to chronic Piezo2 channelopathy and impaired Piezo crosstalk. Indicative of this theory is that activated corneal keratocytes induce nerve growth factor (NGF) production, hence impacting reorganization of nerve fibers in the sub-basal plexi of RA patients [38]. On the contrary, Piezo1 downregulation in activated LCs could result in a functional imbalance in the Th17/Treg ratio, contributing to autoimmunity and DE progress in RA, since it is known that Piezo1 loss leads to increased Treg numbers [39]. Furthermore, Piezo1 upregulation in activated keratocytes due to chronic Piezo2 channelopathy and impaired Piezo crosstalk could also lead to an imbalance in the Th17/Treg ratio, contributing to DED. Overall, impaired somatosensory-terminal Piezo2 and peripheral Piezo1 crosstalk in the cornea of RA-associated DE could result in dysfunctional compensatory upregulated neural regenerative activity. Indeed, Villani et al. found decreased numbers of corneal nerve fibers in RA patients, and they also demonstrated that the tortuosity of these fibers was enhanced and the number of beadlike formations were increased [38]. Villani’s team also detected increased keratocyte activity in the cornea [38], suggesting keratocytes’ active involvement in the immunoregulatory processes.

Indeed, repeated noncontact reinjury of Piezo2-containing somatosensory terminals could lead to chronic Piezo2 channelopathy or sensitization, especially in the presence of underlying genetic and environmental risk factors [16]. Correspondingly, the current authors suggest that the activated K_2P_ channel, through the TASK1 channels, could accelerate this neuroimmune-induced sensitization on the spinal level in autoimmune diseases with concomitant exaggerated LC activation that could also lead to a morphological picture of upregulated regeneration activity of Piezo2-containing somatosensory axons in the cornea. This upregulated regeneration is functionally incomplete as well.

The aforementioned halted and incomplete functional regeneration is associated with a depletory picture in DE and RA not only in terms of the innate and adaptive immune system but in other terms as well. Correspondingly, a recent study demonstrated that a replenishing synthetic tear protein was capable of promoting corneal nerve regeneration even in the presence of associated chronic inflammation via restoring the depleted functional nerve supply [40]. That study was a breakthrough not only because it could provide the first regenerative treatment for DE in the future but because it also highlighted the critical importance of broken and dysfunctional nerve regeneration in the DE disease mechanism.

This is not to mention that the study of Efraim et al. [40] most likely dissected two important pathomechanistic pathways with a potentially effective treatment with Lacripep. The principal pathway seemed to be nerve damage, and chronic ocular inflammation was only secondary. This is in line with the Piezo2 microinjury-induced quad-phasic noncontact injury model of DED, where the primary damage is noncontact mechanoenergetic microdamage of Piezo2-containing corneal somatosensory terminals while only the secondary-damage phase is associated with harsher tissue damage and inflammation [16], meaning that the autoinflammatory pathway must be preceded by mechanoenergetic neuron-terminal microdamage. Noteworthily, that it was postulated that repetitive primary damage could result in sensitization of DED even in the absence of the secondary damage phase [16]. Hence, chronification of the primary damage in the form of the tertiary injury phase, regardless of the existence of secondary damage, is when the repeated microinjury and underlying genetic and environmental risk factors come across in a conflicting way, leading to “kept alive wound healing”, standstill and incomplete functional regeneration and sensitization [16]. Indicative of this theory is that loss-of-function mutation in Piezo2 causes loss of sensitization and pain [41]. Furthermore, the current authors suggest that chronic Piezo2 channelopathy, with resultant impaired Piezo2–Piezo1 crosstalk, not only causes sensitization but concomitant “kept alive wound healing”-induced incomplete functional regeneration, and activated transcription could lead to depletion. Suggestive of this theory is that research is emerging that functionally intact Piezo ion channels indeed act as “molecular breaks” in finishing wound healing in order to safeguard an intimate but complete inflammation process and epithelial integrity reinstatement [42]. Even earlier, it was shown that in psoriasis, another autoinflammatory disease, healing takes more time with Piezo1 than without it [43]. Moreover, involvement of neurons in wound media results in a more sophisticated wound-healing process in corneal epithelial cells [44], indicative of the possible role of Piezo2-containing somatosensory neurons in corneal wound healing [16]. Accordingly, the channelopathy of these Piezo channels could sustain this disruption, therefore impeding due wound closure and wound healing, regardless of the aforementioned K_2P_-TASK1 signaling-axis activation that could further amplify this process in autoimmune diseases.

Efraim et al. emphasized in their paper the essential structural and functional roles of nerves in tissue homeostasis and progenitor cell maintenance [40], as we emphasized the role of Piezo2-containing somatosensory nerves contributing to proprioception in remodeling and regeneration [16]. It is important to note that Piezo1 has a known participatory role in regulation of fate determination of progenitor stem cells [45]. Therefore, the current authors propose that impaired crosstalk between somatosensory-terminal Piezo2 and peripheral Piezo1 due to Piezo2 channelopathy could also cause biased regulation of progenitor stem-cell identity. Accordingly, Lacripep, aside from replenishing depleted transmembrane proteogylcans, may play a central role in restoring functional crosstalk between Piezo2 and Piezo1, hence restoring Piezo1-dependent epithelial progenitor identity and cell fate and, as a result, contributing to completion of wound healing instead of keeping it alive. Noteworthily, according to the altered glycan theory of autoimmunity, every single autoimmune disease has its own glycan profile [46]. Accordingly, the role of proteoglycans in the pathogenesis of RA and resulting proteoglycan-modulating therapies have been the foci of recent research [47]. Furthermore, it is important to note that degradation of proteoglycans biomechanically resembles static compression [48], and this could have molecular implications for indentation and mechanotransduction of piezoelectric channels in the cell membrane. Prolonged or abrupt static compression is indeed implicated in the Piezo2 channelopathy mechanism of the noncontact compression axonopathy theory [8,16,36,49]. Notably, the primary damage of Piezo2 at somatosensory terminals contributing to proprioception was recently proposed to increase the damage of the extracellular matrix (ECM) as part of the secondary injury [50]. Not surprisingly, aggrecan, another proteoglycan, is the first element of the ECM that could be degraded from these compressive forces in cartilage, as well, through faster turnover [51], not to mention that both Piezo1 and Piezo2 contribute to resultant degenerative ECM stiffness [52]. The current authors suggest that this increased stiffness is the result of lost joint-afferent-terminal Piezo2–condrocyte Piezo crosstalk due to the suggested primary damage. It has been long known that degradational cartilage proteoglycan aggrecan epitopes could activate proinflammatory autoreactive T-cell reactions in RA [53,54].

In DE, another answer could be that repetitive ECM damage due to shear stress from tear-film motion and blinking, as well as resultant ECM reorganization, depletes proteoglycans. Indeed, instillation of a chondrocyte-derived ECM facilitated improvement in a mouse model of DE [55]. Hence, replenishing depleted transmembrane and ECM proteoglycans could be one explanation of how Lacripep could restabilize pathologically leaky Piezo channelopathies in somatosensory terminals and re-establish Piezo crosstalk.

Our findings are also interesting in reference to the suggested primary damage of Piezo2-containing somatosensory nerves contributing to proprioception, highlighting that both RA and DED are more prevalent among women [56]. However, this sex difference was not reflected in the abnormal nerve structures in our study. Therefore, the previously proposed sex-specific NGF-TrkA-Piezo2 signaling axis [16] may only contribute to primary pathological hyperexcitatory sensory-terminal microdamage but not to the tertiary injury phase reflected in the corneal morphology studied. Noteworthily, age and disease onset had no impact on the nerve structure either in our examined population.

It is important to emphasize that applying the aforementioned published Piezo microinjury hypotheses and using a multidisciplinary approach in this interpretation of the potential neuroinflammatory pathomechanism in regards to the current abnormal corneal neural structural findings were not meant to challenge any earlier scientific research; on the contrary, they were meant to incorporate it.

One obvious limitation of our investigation is that it was an earlier cross-sectional study, and the reanalysis from a neurocentric view was not part of the original research design. Thus, the results could be considered as preliminary verification of a theory, and further investigation is recommended, with specifically tailored research design and more emphasis on tracing the molecular background of this revealed abnormal neural corneal morphology.

## 4. Materials and Methods

This study was a cross-sectional, comparative study of 40 patients treated, with variable severity of RA, and 44 age- and gender-matched healthy volunteers as control subjects. This study was approved by the Central Ethics Committee of Hungary (ETT TUKEB, 15410-2/2011-EKU). Each participant gave written informed consent about the examinations. All examinations were carried out in accordance with the tenets of the most recent revision of the Declaration of Helsinki.

The RA patients were classified according to the American College of Rheumatology/European League Against Rheumatism (ACR/EULAR, Zürich, Switzerland) criteria [57]. The DAS28 was calculated at the time of examination. Individuals with any overt ocular symptom requiring specific ophthalmic assistance; diabetes; previous eye surgery; uveitis within one year prior to examination; glaucoma; congenital, mechanical or toxic injury of the cornea; or illness causing corneal edema, haze or scars were excluded. RA patients with secondary Sjögren’s syndrome, diagnosed according to the revised version of the European criteria for Sjögren’s syndrome, were also excluded, since it has been described that Sjögren-related hypolacrimation results in severe dry eye, which itself results in significant changes in the morphology of the sub-basal nerve plexus (SBNP) [58,59]. Patients with Schirmer test results of less than 5 mm/5 min were not excluded automatically from this study if the results of other functional tests and the histology were normal and no anti-Ro (SSA) or anti-La (SSB) antibodies were detected.

First, dry-eye-related signs and symptoms were evaluated. Assessment of subjective ocular discomfort related to DE symptoms was performed using the OSDI questionnaire to evaluate subjective ocular discomfort [60]. The study participants went through a conventional slit-lamp biomicroscopic examination. A Schirmer strip (Haag-Streit, Köniz, Switzerland) was used to evaluate tear production. The strip was placed into the temporal part of the lower cul-de-sac without anesthesia, and values were read after 5 min. TBUT was determined by staining the ocular surface with fluorescein vital dye and represented the interval between the last complete blink and the first appearance of a corneal black spot, in seconds. The mean value of three consecutive measurements was interpreted as the TBUT.

Then, in vivo corneal laser-scanning confocal microscopy was performed to describe the morphology of the SBNP. All patients were examined with the Rostock Cornea Module of Heidelberg Retina Tomograph-III (Heidelberg Engineering GmbH, Heidelberg, Germany) equipped with the built-in software Heidelberg Eye Explorer version 1.5.10.0. Before the examination, one drop of oxybuprocaine hydrochloride (Oxybuprocaine Humacain 0.4%, Human Pharmaceuticals, Gödöllő, Hungary) was instilled topically. The microscope lens was covered by a sterile-packaged disposable polymethyl methacrylate cap (TomoCap^®^, Heidelberg Engineering GmbH) to keep a stable distance between the cornea and the microscope head. One drop of artificial tear gel (0.2% carbomer, Vidisic^®^, Chem.-pharm. Fabrik GmbH, Brunsbütteler, Berlin, Germany, Bausch&Lomb) was used as a coupling medium to ensure airless contact. Two-dimensional en face images were taken of the SBNP in the central cornea using a 400 µm field-of-view lens. All examinations were carried out in the same room under unchanged conditions.

ACCMetrics V.2 software was used to evaluate the morphometric parameters of the SBNP [61]. A set of images (between 5 and 10) representing the SBNP was collected, and the following parameters were automatically quantified by the software using multiple analysis mode: CNFD; CNFL; CTBD and corneal nerve-fiber width (CNFW, the average nerve-fiber width in mm/mm^2^).

Variables of the selected sample were presented as means and standard deviations. For the purpose of selecting the adequate statistical procedure, the Shapiro–Wilk W test of normality was calculated. For comparison of the measured datasets, an independent sample t-test and a Mann–Whitney U test were performed. To further support the results of this study, we calculated and included effect-size (ES-Cohen’s d) values. For the correlation analysis, Pearson’s parametric and Spearman’s nonparametric calculations were performed. Statistical calculations were executed with Statistica software (TIBCO Software Inc. Palo Alto, CA, USA, installed version 14.0.0.15) and JASP software (jasp-stats.org, installed version 0.16). The significance level was determined in all cases to be *p* < 0.05.

## 5. Conclusions

The results of our confocal microscopy study demonstrated that indeed, corneal sensory afferents show reduced regeneration activity with underlying damaged nerve structure in DE secondary to RA. All investigated parameters were significantly lower in the RA patients compared to the control samples, such as the number of fibers, the total length of the nerves, the number of branch points on the main fibers, and the total nerve-fiber area. The authors of this manuscript propose that the observed corneal sensory morphology could have been the result of impaired crosstalk between corneal somatosensory-terminal Piezo2 and peripheral-cell Piezo1 due to chronic Piezo2 channelopathy. Moreover, the authors theorized that this corneal somatosensory-terminal Piezo2 channelopathy could evolve due to even noncontact mechanoenergetic corneal sensory-afferent damage crosslinked with underlying RA-derived spinal K_2P_-TASK1 signaling-axis activation. In summary, the referenced chronic corneal sensory-terminal Piezo2 channelopathy could result in the observed broken and dysfunctional regeneration activity in association with the theorized activated transcription, impaired Piezo2–Piezo1 crosstalk, activated LCs, activated corneal keratinocytes and breaks in the wound-healing process, not to mention a Th17/Treg imbalance in the neuroinflammatory link.

## Figures and Tables

**Table 1:** Demographic and clinical data in the control and rheumatoid arthritis (RA) groups. Data are shown in mean, SD and min–max values; disease activity score (DAS28); C-reactive protein (CRP); tear break-up time (TBUT); and ocular-surface disease index (OSDI). * *p* < 0.05 Mann–Whitney test.

	Control	RA
No. of Patients	44	40
No. of Eyes	44	53
Age (Years)	45.9 ± 20.2 [19–73]	50.6 ± 13.2 [28–74]
Gender (Male/Female)	20/24	13/27
RA Duration (Years)	-	10.5 ± 9.2 [0.3–53]
DAS28	-	2.6 ± 0.9 [0.9–5.1]
CRP	-	5.9 ± 6.5 [0.1–30.5]
TBUT (s)	11.3 ± 3.0 [4–16] *	8.9 ± 3.6 [4–20]
Schirmer Test (mm/5 min)	12.2 ± 3.0 [5–17] *	6.9 ± 5.8 [0–26]
OSDI	10.1 ± 8.3 [0–35]	34.2 ± 25.2 [0–94] *

**Table 2:** Mean and SD values for the CNFD–CNBD–CNFL–CTBD–CNFA variables of the RA and control samples, with *p* values and Cohen’s d values of the comparisons.

	Group	N	Mean	SD	*p*	Cohen’s d
CNFD ^1^ Average	RA	52	13.92	6.69	0.001	0.749
	Control	39	19.02	6.98		
CNBD ^2^ Average	RA	52	15.32	9.04	0.006	0.722
	Control	39	23.45	13.69		
CNFL ^3^ Average	RA	52	10.57	3.22	0.002	0.777
	Control	39	13.07	3.22		
CTBD ^4^ Average	RA	52	30.03	15.35	0.01	0.61
	Control	39	40.21	18.31		
CNFA ^5^ Average	RA	52	0.0053	0.0021	0.007	0.533
	Control	39	0.0063	0.0018		
CNFD Highest	RA	52	2000	10.71	0.018	0.481
	Control	39	25.09	10.43		
CNBD Highest	RA	52	27.04	20.70	0.044	0.499
	Control	39	38.94	27.48		
CNFL Highest	RA	52	14.53	4.16	0.014	0.617
	Control	39	17.14	4.33		
CTBD Highest	RA	52	47.83	31.94	0.089	0.387
	Control	39	60.89	35.95		
CNFA Highest	RA	52	0.0072	0.0031	0.087	0.27
	Control	39	0.0798	0.0026		

^1^ CFND: the number of fibers per mm^2^; ^2^ CFNBD: the number of branch points on the main fibers per mm^2^; ^3^ CNFL: the total length of nerves in mm per mm^2^; ^4^ CTBD: the number of branch points per mm^2^; ^5^ CNFA: the total nerve-fiber area in mm^2^ per mm^2^.

**Table 3:** Correlation coefficient values for the variable pairs in the correlations. For all cases, correlation was significant (*p* < 0.05).

Variable	r
CNFD Average—Schirmer	0.339
CNFL Average—Schirmer	0.306
CNFD Highest—Schirmer	0.340
CNFA Highest—CRP	−0.328

## Data Availability

The data presented in this study are available on request from the corresponding authors.

## Abbreviations

CNBD	Corneal nerve-branch density
CNFA	Corneal nerve-fiber area
CNFD	Corneal nerve-fiber density
CNFL	Corneal nerve-fiber length
CNFW	Corneal nerve-fiber width
CTBD	Corneal nerve-fiber total branch density
CRP	C-reactive protein
DAS28	Disease activity score
DE	Dry eye
DED	Dry eye disease
ECM	Extracellular matrix
Hsp70	Heat shock protein 70
IL-6	Interleukin-6
IL-17	Interleukin-17
LC	Langerhans cells
MS	Multiple sclerosis
NGF	Nerve growth factor
NKT cell	CD3^+^/CD56^+^ natural killer-T cell
NF-κB	Nuclear factor kappa B
OSDI	Ocular-surface disease index
RA	Rheumatoid arthritis
SBNP	Sub-basal nerve plexus
TASK1	TWIK-related acid-sensitive K^+^ channel 1
TBUT	Tear break-up time
Th1	CD4^+^IFN-γ^+^
Th2	CD4^+^IL-4^+^
Th17	CD4^+^IL-17^+^
TLR4	Toll-like receptor 4
TNF-α	Tumor necrosis alpha
Treg	T regulatory cell
